# Comprehensive analysis of prognostic characteristics based on T cell-mediated tumor killing related genes in triple negative breast cancer

**DOI:** 10.3389/fimmu.2026.1801004

**Published:** 2026-04-10

**Authors:** Chenyu Zhang, Yanmin Hu, Yongming Han, Peipei Zhao, Baosan Han, Xingjie Hu

**Affiliations:** 1 Department of Breast Surgery, Xinhua Hospital Affiliated of Shanghai Jiaotong University School of Medicine, Shanghai, China; 2 Research Center of Breast Tumor Intelligent Diagnosis and Treatment, University of Shanghai for Science and Technology, Shanghai, China; 3 Department of Gerontology, The Second Affiliated Hospital of Zhengzhou University, Zhengzhou, Henan, China; 4 State Key Laboratory of Systems Medicine for Cancer, Shanghai Cancer Institute, Renji Hospital, Shanghai Jiao Tong University School of Medicine, Shanghai, China; 5 Micro-Nano Research and Diagnosis Center, Renji Hospital, School of Medicine, Shanghai Jiao Tong University, Shanghai, China; 6 Department of Nephrology, Molecular Cell Lab for Kidney Disease, Shanghai Peritoneal Dialysis Research Center, Ren Ji Hospital, Uremia Diagnosis and Treatment Center, Shanghai Jiao Tong University School of Medicine, Shanghai, China; 7 State Key Laboratory of Oncogenes and Related Genes, Center for Single-Cell Omics, School of Public Health, Shanghai Jiao Tong University School of Medicine, Shanghai, China

**Keywords:** gene set enrichment analysis, immune microenvironment, prognostic model, T cell-mediated tumor killing, triple-negative breast cancer

## Abstract

**Background:**

Triple-negative breast cancer (TNBC) is an aggressive subtype with high malignancy and poor prognosis. Immunotherapy is a promising treatment for TNBC patient. Although T cell-mediated tumor killing related genes (TTKRGs) play critical roles in antitumor immunity, their prognostic value and potential function in TNBC is still unclear.

**Methods:**

Transcriptomic data from TCGA-BRCA and TTKRGs were curated to determine the prognostic genes in TNBC and a prognostic model was further established. GSE135565 dataset was used to validate the prognostic model. Furthermore, the differences between risk groups were compared through ESTIMATE, clinical correlation, drug sensitivity, immune checkpoint, tumor microenvironment. GSEA and GeneMANIA analysis were performed to explore the potential mechanism.

**Results:**

Intersection of 1,933 differentially expressed genes (DEGs) and 1,109 TTKRGs yielded 88 candidate genes, and PODN, SEMA7A, GPR34, and COCH were identified as prognostic genes for TNBC. A prognostic model was further successfully established and validated. The model exhibited good predictive performance in both training and validating sets with AUC values all above 0.6. Our studies confirmed the pathological stages were associated with risk scores and there were significant differences in the drug sensitivity, immune checkpoint expression, and tumor microenvironment among different risk groups. The two groups were enriched in pathways of cell cycle and immune regulation and the four prognostic genes were associated with transcription factors such as SP1, MYC, and CTCF.

**Conclusion:**

We constructed a robust prognostic model based on four T cell-mediated tumor killing (TTK)-related genes. Beyond predicting survival, this signature effectively decodes the immunosuppressive tumor microenvironment (TME) in TNBC, characterized by stromal activation, M2 macrophage polarization, and T cell exhaustion. These findings highlight novel immune evasion mechanisms and provide a theoretical foundation for targeting next-generation immune checkpoints and specific stromal-immune crosstalk in TNBC immunotherapy.

## 1 Introduction

As an aggressive subtype, triple-negative breast cancer (TNBC) lacks of effective receptors of conventional hormone therapy and targeted therapy. TNBC is correlated with a higher probability of recurrence and distant metastasis, which leads to a lower survival rate (1). Systemic chemotherapy, including paclitaxel, anthracycline, and/or platinum based therapies, is the first-line treatment choice for advanced TNBC. However, some patients who received chemotherapy have a bad prognosis (2, 3). Notably, the development of immunotherapy in the past years, such as immune checkpoint inhibitors (ICIs), has changed the treatment paradigm for TNBC (2). Nevertheless, a large number of TNBC patients fail to demonstrate a good response to ICIs or develop acquired resistance, posing a big challenge for TNBC immunotherapy (4). Exploring the pathogenesis of TNBC in depth, identifying predictive biomarkers to assist in selecting treatment methods, developing new treatment combinations to combat drug resistance, and refining treatment targets have become urgent issues that need to be addressed.

As is known, T cell-mediated tumor killing (TTK) plays an important role in the immunotherapy. Antigen-presenting cells, like dendritic cells, capture and process tumor-derived antigens and then present to naïve T cells. Once T cells receive essential co-stimulatory signals, they get activated, expand clonally, and differentiate into effector T cells (5, 6). These cytotoxic T lymphocytes then traffic to the tumor site, where they recognize and eliminate malignant cells by releasing cytotoxic granules or engaging death receptor pathways, thereby executing precise tumor cell killing (7). The pivotal role of TTK is particularly prominent in TNBC. Compared to other subtypes, TNBC commonly displays a higher tumor mutational burden and richer tumor infiltrating lymphocytes (TILs), potentially fostering a more robust TTK response (8, 9). The presence and extent of TILs have emerged as significant positive prognostic biomarkers in TNBC. Clinically, the ICIs therapy, like PD-L1 antibody is designed to enhance TTK by blocking the PD-1/PD-L1 inhibitory pathway, thereby reactivating suppressed antitumor immunity and significantly improving patient outcomes (10). However, in clinical practice, a main concern for choosing ICIs is the low response rate. The objective response rate (ORR) of TNBC to ICIs is only about 5-10% and about 10-20% in PD-L1 positive patients, making clinical trial results not as potent as expected (11). This limited efficacy is largely attributed to the complex and highly immunosuppressive tumor microenvironment (TME) of TNBC, which actively establishes physical and biochemical barriers to impede T cell infiltration and orchestrates T cell exhaustion. Consequently, the sensitivity of tumor cells to T cell-mediated killing (TTK) is dynamically restricted by both intrinsic tumor signaling and extrinsic stroma-immune crosstalk. Therefore, decoding the specific genes that govern TTK resistance and TME remodeling is urgently needed to break immune tolerance and discover next-generation immunotherapeutic targets beyond the conventional PD-1/PD-L1 axis.

Additionally, the sensitivity and resistance of tumor cells to T cell-mediated killing is dynamically regulated by multiple pathways in tumor cells. For example, it is reported that autophagy defect could prevent T cell-mediated tumor killing. It is found that in autophagy-impaired TNBC cells, the restraint of Tenascin-C makes T cell mediated tumor killing more responsive and enhances the antitumor effects of single anti-PD1/PDL1 therapy (12). Ru et al. also revealed that PTPN2 and CD47 affect the response of tumor cells to T cell-mediated killing (13). In light of the above research, T cell-mediated tumor killing related genes (TTKRGs) matter a great deal in tumor immunotherapy. The prognostic value of using TTK sensitivity related genes to distinguish subtypes with different immune phenotypes and immune cell infiltration characteristics has been evaluated (14–16). Nevertheless, TTK-related features in TNBC are still limited in predicting the immunotherapy response and efficiency. Thus, in order to improve the clinical role of TTK-related features, further exploration is needed. So, it’s necessary to further investigate the mechanisms of TTK in TNBC, so as to provide new therapeutic avenues and improve clinical prognoses for TNBC patients.

Herein, we integrated the transcriptomic data from public databases and TTKRGs to screen candidate genes, and prognostic genes associated with TNBC survival were identified by univariate Cox regression analysis. Subsequently, based on prognostic genes expression levels, a prognostic model was established by random survival forest. The prognostic model was further validated in both training and validation sets, which achieved satisfactory prediction with AUC values all above 0.6. Furthermore, we investigated clinical characteristics, drug sensitivity, and tumor immune infiltration of different risk groups to study the internal mechanisms. The results provide a new theoretical rationale for elucidating the pathophysiological mechanism of TNBC and for the targeted treatment of TNBC.

## 2 Materials and methods

### 2.1 Data collection

For training set, TCGA-BRCA dataset was derived from the TCGA database (https://portal.gdc.cancer.gov/). This dataset consisted of 114 TNBC samples (108 with survival information) and 99 normal breast samples. All data were derived from high-throughput sequencing and used for differential expression analysis and prognosis model. For external validation set, dataset GSE135565 included 84 TNBC samples and its clinical information (sequencing platform: GPL570) were obtained from the Gene Expression Omnibus (https://www.ncbi.nlm.nih.gov/geo/). Furthermore, 1109 TTKRGs were obtained from the TISIDB (http://cis.hku.hk/TISIDB) (**Supplementary Table 1**).

### 2.2 Identification of differentially expressed genes and candidate genes

Differential expression analysis in the training set was carried out by “DESeq2” package (v 1.42.0) (17) to identify DEGs. DEGs were screened with the thresholds |log_2_ fold change (FC)| > 2 and p < 0.05. Furthermore, the “ggplot2” package (v 3.5.1) (18) was utilized to drew a volcano plot. A heatmap was drawn by the “ComplexHeatmap” package (v 2.21.1) (19) showing the top 5 most significantly up- and down- DEGs. Then, the “VennDiagram” package (v 1.7.3) (20) was used to identify candidate genes.

### 2.3 Pathway enrichment and protein-protein interaction analysis

The Gene Ontology (GO) and Kyoto Encyclopedia of Genes and Genomes (KEGG) pathways were determined based on “clusterProfiler” package (v 4.7.1.3) (21) (p < 0.05). The top 5 enriched pathways from GO categories of molecular function (MF), cellular component (CC) and biological process (BP) and the top 10 enriched pathways of KEGG analysis were selected. PPI analysis was conducted by the candidate genes utilizing the information from Search Tool for the Retrieval of Interacting Genes/Proteins (STRING) platform (https://www.string-db.org/) with an interaction score threshold > 0.150. Isolated gene nodes without protein-protein interaction with other genes were removed. Finally, the PPI network was visualized through “circlize” package (v 0.4.16) (22).

### 2.4 Construction of prognostic model

To identify prognostic genes, univariate Cox regression analysis was employed for each candidate genes based on the training set samples using the “survminer” package (v 0.4.9) (23). The proportional hazards (PH) assumption test was then performed by ggcoxzph function from the “survival” package (v 3.7.0) (23) to verify these genes with PH assumption (p > 0.05). The “randomForestSRC” package (v 3.2.3) (24) was utilized to construct a random survival forest (RSF) model based on prognostic genes. The number of trees (ntree) and number of split variables (mtry) were determined to calculate risk score. The importance ranking of the prognostic genes was also derived from the model.

Further, a risk model formula was developed based on expression levels of the prognostic genes and overall survival (OS) information of samples in the training set. Risk score for each sample was calculated as follows:


$$\text{risk score }=\;\sum_{\text{i}=1}^{\text{n}}(\text{coef}({\text{gene}}_{\text{i}})\;\times \text{ expr}({\text{gene}}_{\text{i}}))$$

Based on the optimal risk score cutoff and minprop parameter, TNBC patients were grouped into high- and low-risk groups. The prognostic model was then assessed with risk score distribution diagram and survival status diagram by the “ggplot2” package (v 3.5.1). Kaplan-Meier (K-M) curves were created through “survminer” package (v 0.4.9) to investigate survival differences. Time-dependent receiver operating characteristic (ROC) curves were generated by “survivalROC” package (v 1.0.3.1) (25) at 3, 5, and 7 year. The area under the curve (AUC) was calculated to determine the predictive accuracy.

### 2.5 Clinical correlation analysis of TNBC

TNBC patients in training set were further grouped according to different clinical features (age, clinical stage, T, N, M). Then, the Wilcoxon rank-sum test was utilized to analyse the differences of risk scores between different clinical features subgroups of TNBC patients. Moreover, the survival differences were compared between different clinical subgroups.

### 2.6 Drug sensitivity analysis of TNBC

198 chemotherapeutic agents were obtained from the Genomics of Drug Sensitivity in Cancer (GDSC, http://www.cancerrxgene.org/) database. The “oncoPredict” package (v 1.2) (26) and rank-sum test were utilized to calculate and compare the half-maximal inhibitory concentration (IC50) values.

### 2.7 ESTIMATE and immune checkpoints analysis of TNBC

Then, the ESTIMATE algorithm was utilized to estimate the immune, stromal, and ESTIMATE scores. The rank-sum test was used to analyse the three scores and immune checkpoint expression between groups.

### 2.8 Immune microenvironment of TNBC

To explore the immune infiltration of TNBC, ssGSEA algorithm in “GSVA” package (v 1.50.0) (27) was applied to TNBC samples with complete survival information in the training cohort. The Spearman correlation coefficients between any two differentially enriched immune cells and between prognostic gene and differentially enriched immune cell were calculated by the “psych” package (v 2.4.3) based on TNBC samples with survival information in the training set (|cor| > 0.3, p < 0.05).

### 2.9 Chromosomal localization and subcellular localization

The “RCircos” package (v 1.2.2) (28) was used to visualize the distribution of prognostic gene on each chromosome. The subcellular localization information of these genes was obtained from the GeneCards database (https://www.genecards.org/) to explore the potential mechanism of these genes.

### 2.10 Gene set enrichment analysis and GeneMANIA analysis

The “c2.cp.kegg.v7.4.symbols.gmt” was downloaded from MSigDB (https://www.gsea-msigdb.org/gsea/msigdb/) as reference gene set. Differential analysis between risk groups in TNBC samples from training cohort was performed and log2FC values were calculated. Then, the GSEA was conducted using the “clusterProfiler” package (v 4.7.1.3) (p < 0.05, |normalized enrichment score (NES)| > 1). Further, we submitted these genes to GeneMANIA database (https://genemania.org/) to identify genes functionally similar to the prognostic genes. Finally, top 20 genes with highest functional similarity scores were chosen to construct interaction network.

### 2.11 Construction of molecular regulatory network

Transcription factors (TFs) associated with prognostic genes were obtained from the miRNet database (https://www.mirnet.ca/). A TF-mRNA regulatory network was subsequently generated by Cytoscape software (v 3.10.2) (29).

### 2.12 Cells and animals

The 4T1 and MDA-MB-231 cell lines were purchased from the Chinese Academy of Sciences Cells Bank (Shanghai). Female Balb/c mice (aged 6 weeks) were purchased and housed in the Animal Research Center of Renji Hospital. All animal care and relevant experimental procedures were approved by RenJi Hospital Ethics Committee and performed strictly according to the guidelines of China Animal Welfare Legislation.

### 2.13 Cell transfection

Lipofectamine^®^ 2000 (ThermoFisher, USA) was used for the transfection of SEMA7A siRNA (si-SEMA7A) and negative control siRNA (si-NC) in MDA-MB-231 and 4T1 cells. The siRNA sequences used are shown in **Supplementary Table 2**.

### 2.14 Quantitative real-time PCR

Total RNA were isolated using RNA extraction kit (Yeasen Biotechnology, China) and then reverse transcribed with a reverse transcription reagent kit (TaKaRa, Japan). Then, the qPCR experiment was performed with SYBR Premix Ex Taq kit (TaKaRa, Japan) and the relative mRNA expression levels were analysed. The primer sequences used are shown in **Supplementary Table 3**.

### 2.15 Western blotting

After different treatments, the total protein samples were prepared and quantified by a BCA protein assay kit. Then, the proteins were separated by SDS - PAGE and transferred to PVDF membranes, which were further blocked in 5% bovine serum albumin (BSA) for 1 h and incubated with antibodies (SEMA7A Polyclonal antibody, Proteintech, 18070-1-AP, China) overnight at 4 °C. After 1h incubation with the secondary antibody (ThermoFisher, A-11008, USA), the protein bands were visualized using Odyssey CLx Imager (Licor, 9140-00, USA).

### 2.16 Cell proliferation assay

MDA-MB-231 and 4T1 cells were cultured in 96-well plates. Then, at certain time points (0, 24, 48, and 72 h), 10 µL CCK-8 solution was added and incubated for 2 h. Then, the absorbance of each wall was measured by a microplate reader at 450 nm.

### 2.17 Wound healing assay

After transfection, the cells were cultured in 6-well plates until the cells were confluent. Then use a pipette tip to scratch on 6-well plates and create the wound paths. Next, the cells were incubated with medium without FBS for 24 h. We photographed and measured all the wounds at the beginning (0 h) and 24 h after the scratch.

### 2.18 Mouse model of orthotopic breast tumor

Firstly, we anesthetized mice with pentobarbital. Then a small incision was made between the fourth nipple. A tweezer was used to fully squeeze and expose the mammary fat pad from base. After that, 1×10^6^ 4T1 cells were injected into the mammary fat pad gently. The injection site was pressed for at least 30 s using cottons wabs to prevent the leakage of cell suspensions, and the skin incision was sutured by 3 surgical knots. The following experiments were carried out when the tumor size reached about 100mm^3^. Tumor volumes were measured every two days to track the tumor growth. si-SEMA7A and si-NC were intratumoral injection every three days, respectively. Tumors were harvested and measured after a 14-day treatment period. In this study, according to ethical requirements, if the maximum diameter of the tumor exceeded 15mm or the tumor metastasized or rapidly grew to ulceration, causing infection or necrosis, the mice were euthanized. We confirmed that throughout the entire duration of this study, the maximal tumor burden was not exceeded.

### 2.19 Statistical analysis

R software (v 4.2.2) was used for all analyses. Wilcoxon test was used to analyse differences between high- and low-risk groups. To compare the si-SEMA7A and si-NC groups, we used the student’s t-test. p-values <0.05 was considered statistically significant.

## 3 Results

### 3.1 Identification and function analysis of candidate genes

**Figure 1:**
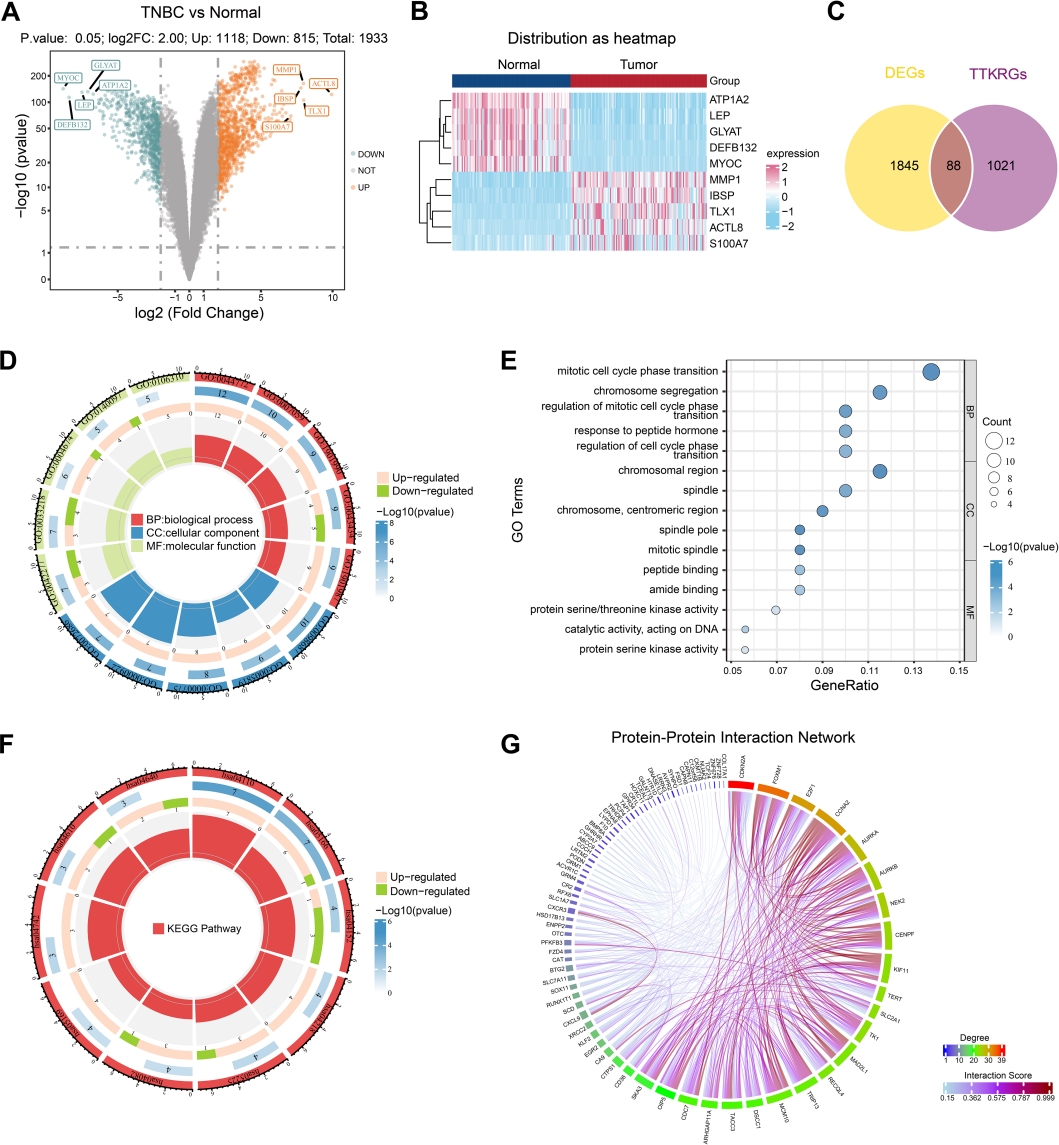
Investigation of candidate genes. **(A)** Volcano plot of 1933 differentially expressed genes between TNBC and normal samples from TCGA-BRCA dataset. **(B)** The expression heatmap of the top 5 up-regulated and top 5 down-regulated DEGs. **(C)** Venn diagram visualizing the intersections between DEGs and TTKRGs. **(D)** GO analysis circle plot displaying gene annotation enrichment analysis of the 88 candidate genes. **(E)** GO enrichment analysis of the top 5 enriched terms in 351 BPs, 33 CCs, and 39 MFs. **(F)** KEGG analysis circle plot of the 88 candidate genes. **(G)** PPI network of the 88 candidate genes.

A total of 1,933 DEGs were identified between TNBC and normal samples. Among them, there were 1,118 up-regulated and 815 down-regulated genes in TNBC group. The top 5 up-regulated DEGs were ACTL8, TLX1, MMP1, IBSP, and S100A7 and the top 5 down-regulated DEGs included MYOC, DEFB132, LEP, GLYAT, and ATP1A2 (**Figures 1A, B**). Intersection analysis between the 1,933 DEGs and 1,109 TTKRGs yielded 88 candidate genes (**Figure 1C**). GO enrichment analysis showed the 88 candidate genes were significantly enriched in 351 BPs, 33 CCs, and 39 MFs. Among BPs, the most enriched term was mitotic cell cycle phase transition. In CCs, the most enriched term was chromosomal region. For MFs, the most enriched term was peptide binding (p < 0.05) (**Figures 1D, E**, **Supplementary Table 4**). KEGG enrichment analysis identified 16 significantly enriched pathways, such as Cell cycle, AMPK signaling pathway, Hepatocellular carcinoma (p < 0.05) (**Figure 1F**, **Supplementary Table 5**). The PPI network consisted of 80 protein nodes and 448 interaction edges after removing eight isolated gene nodes that lacked protein-protein interactions with other genes. The most highly connected genes included CDKN2A, FOXM1, E2F1, CCNA2, and AURKA (**Figure 1G**).

### 3.2 Construction and validation of prognostic models

**Figure 2:**
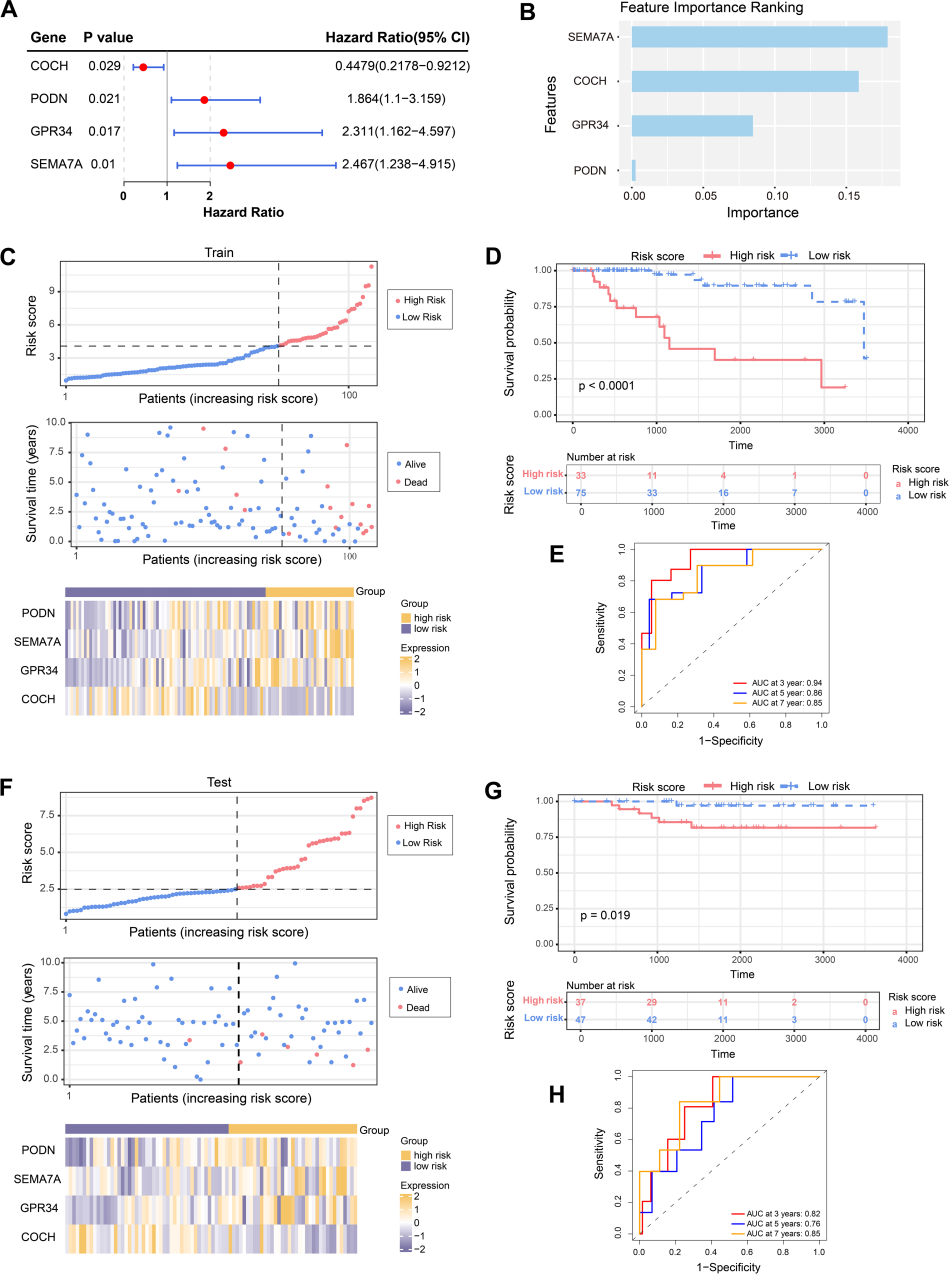
Construction and validation of prognostic model. **(A)** Forest plot of the univariate Cox regression analysis results of prognostic genes. **(B)** The importance ranking of the prognostic genes derived from the model. **(C)** Risk score distribution, survival status, and expression of the prognostic genes of the low-risk and high-risk groups in the training set. **(D, E)** K-M survival analysis **(D)** and ROC analysis **(E)** of the survival rates of patients in the training set. **(F)** Risk score distribution, survival status, and expression of the prognostic genes of the low-risk and high-risk groups in the validation set. **(G, H)** K-M survival analysis **(G)** and ROC analysis **(H)** of the survival rates of patients in the validation set.

Through univariate Cox regression analysis, we identified four candidate genes (PODN, SEMA7A, GPR34, and COCH) (p < 0.05) (**Figure 2A**). PODN, SEMA7A, and GPR34 were identified as risk factors (HR > 1, p < 0.05). COCH was identified as a protective factor (HR < 1, p < 0.05). These four genes also satisfied the PH assumption and were confirmed as prognostic genes (p > 0.05) (**Supplementary Figure 1**). In the RSF model, the variable importance ranking of the prognostic genes was SEMA7A > COCH > GPR34 > PODN (**Figure 2B**). The training set samples were further grouped into high-risk (n = 33) and low-risk (n = 75), using the optimal cutoff value (4.082561) and a minimum proportion (minprop) of 0.3. Furthermore, the risk score distribution and survival status analysis confirmed a poorer survival outcome in high-risk group. Expression heatmap revealed that PODN, SEMA7A, and GPR34 were highly expressed in the high-risk group, and COCH was highly expressed in the low-risk group (**Figure 2C**). Besides, K-M curve analysis confirmed that the survival rate of high-risk groups was lower (**Figure 2D**). ROC curve analysis also confirmed this prognostic model showed satisfactory performance in predicting patient survival, with AUC values for 3, 5, and 7year survival predictions all above 0.6 (**Figure 2E**).

During validation of the prognostic model, patients in the validation set were grouped into high-risk (n = 37) and low-risk (n = 47) with the optimal risk score cutoff (2.490559) and a minimum proportion (minprop) of 0.1. Furthermore, the risk score distribution and survival status analysis indicated a higher number of deaths in the high-risk group. This external validation results indicating that the prognostic model has good generalizability. Similarly, the expression heatmap showed that PODN, SEMA7A, and GPR34 were highly expressed in the high-risk group, while the low-risk group had higher expression levels of COCH (**Figure 2F**). K-M curve analysis further confirmed that the high-risk patients exhibiting poorer survival outcomes (**Figure 2G**). Besides, ROC curve analysis also demonstrated the good prognostic predictive capability with AUC values for 3-, 5-, and 7-year survival predictions all exceeded 0.6 (**Figure 2H**).

### 3.3 Clinical relevance and drug sensitivity analysis

**Figure 3:**
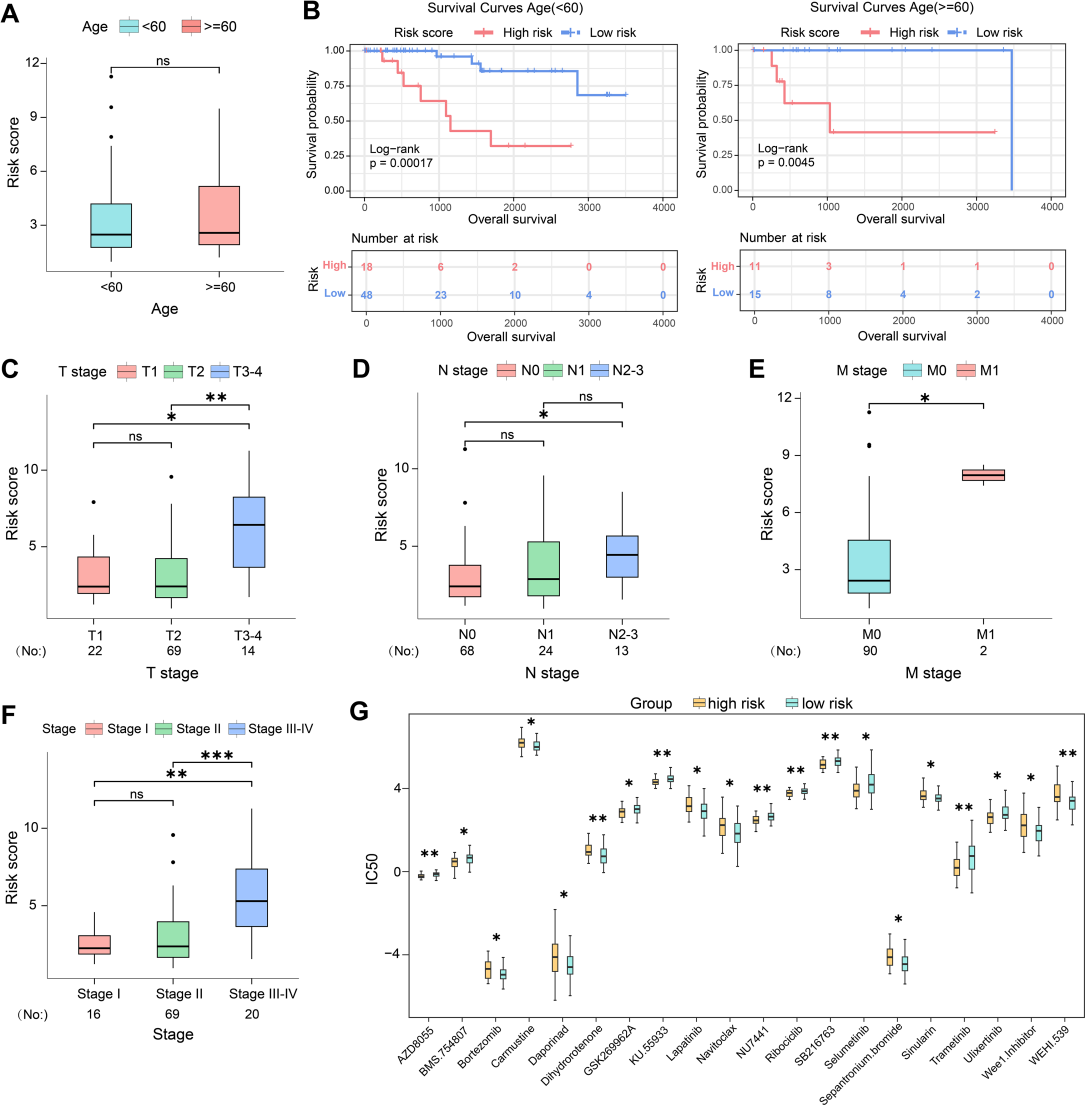
Associations between risk scores and clinicopathological characteristics. **(A)** Correlation analysis between risk score and different age. **(B)** Survival differences between high-risk and low-risk groups in different age subgroups. **(C)** Correlation analysis between risk score and different T stage. **(D)** Correlation analysis between risk score and different N stage. **(E)** Correlation analysis between risk score and different M stage. **(F)** Correlation analysis between risk score and different pathological stage. **(G)** Drug sensitivity analysis of the IC50 distribution of chemotherapy drugs between high- and low-risk TNBC groups. Statistical analysis was performed with Log-rank test and Wilcoxon test. *p < 0.05, **p < 0.01, ***p < 0.001.

Furthermore, the high- and low-risk groups were further stratified based on age (**Figures 3A, B**). The K-M curve analysis indicated the high-risk patients exhibiting poorer survival outcomes in both age<60 and age>=60 subgroups (**Figure 3B**). Clinical correlation analysis also confirmed that pathological stage, as well as T, N, and M stages, were significantly associated with risk score stratification among subgroups (p < 0.05) (**Figures 3C–F**). Besides, the two risk groups showed significant differences in drug sensitivity analysis. Higher IC50 values of 25 compounds were confirmed in the high-risk group, suggesting reduced drug sensitivity. The top 20 compounds including AZD8055 (mTOR kinase inhibitor), BMS-754807 (IGF-1R/IR inhibitor) and bortezomib (proteasome inhibitor) were shown in **Figure 3G**. The reduced drug sensitivity of high-risk group may be due to the fact that in addition to direct cytotoxicity, these drugs can also exert immune regulatory functions that influence treatment response. For example, mTOR inhibitors can enhance antitumor memory lymphocytes, potentially remodeling the tumor immune microenvironment (30).

### 3.4 ESTIMATE, immune checkpoints and immune microenvironment analysis

**Figure 4:**
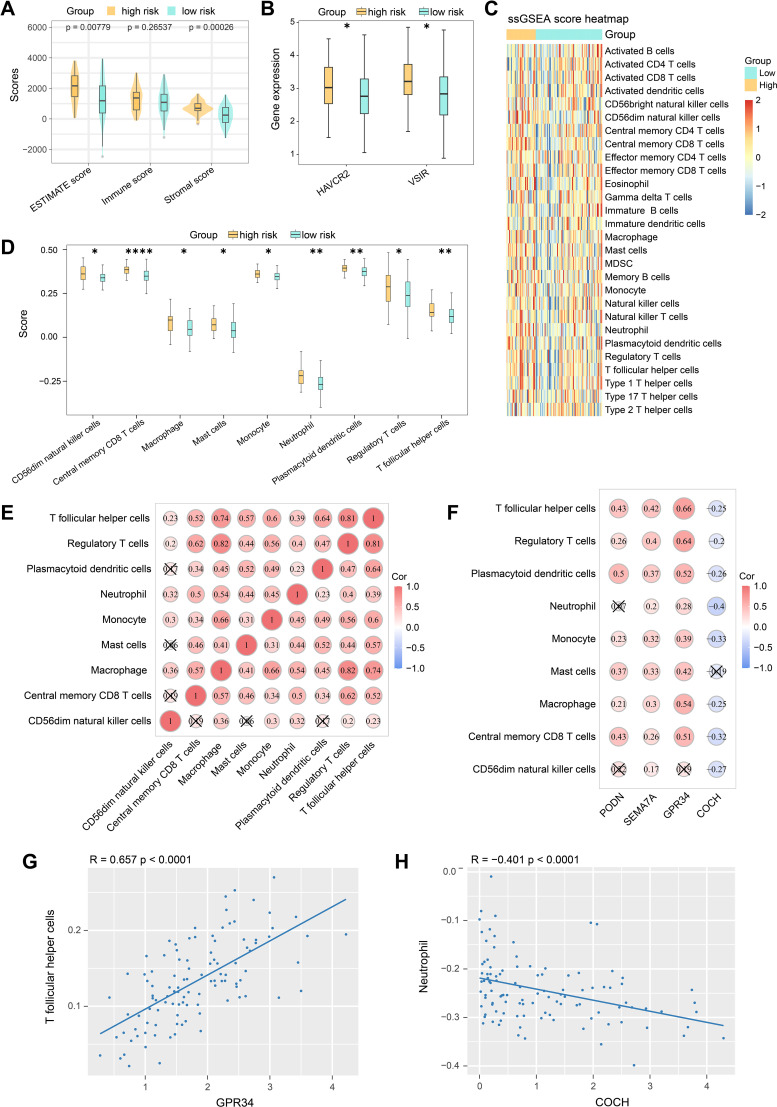
Associations between risk scores with tumor micro-environment and immune cell infiltration. **(A)** Differences in ESTIMATE score, stromal score and immune score between high- and low-risk TNBC groups. **(B)** Differences of immune checkpoint expression between high- and low-risk TNBC groups. **(C)** The ssGSEA score heatmap depicting the enrichment scores of 28 immune cell types in TNBC high- and low-risk groups. **(D)** Differences of immune cells between high- and low-risk TNBC groups. **(E)** Correlation analysis among differential immune cells. **(F)** Correlation analysis among differential immune cells and prognostic genes. **(G)** Correlation between the GPR34 expression and the infiltration levels of T follicular helper cells. **(H)** Correlation between the COCH expression and the infiltration levels of neutrophils. Statistical analyses were performed with Wilcoxon test. *p < 0.05, **p < 0.01, ****p < 0.0001.

ESTIMATE analysis showed that both ESTIMATE and stromal scores of the high-risk group were significantly higher than the low-risk group, indicating distinct composition of stromal and immune cells within the tumor microenvironment (TME) between the two risk groups (**Figure 4A**). Specifically, the high-risk group exhibited a distinct ‘immunosuppressive stromal activation’ phenotype. These elevated scores suggest that the TME in high-risk patients is characterized by dense desmoplasia, which typically acts as a physical barrier restricting the penetration and activation of cytotoxic T lymphocytes (CTLs). Furthermore, immune checkpoints expression analysis was conducted and the results showed higher expression levels of HAVCR2 and VSIR in the high-risk group (**Figure 4B**). Significantly, the concurrent upregulation of these next-generation immunosuppressive checkpoints, HAVCR2 (TIM-3) and VSIR (VISTA), strongly indicates a state of severe T cell exhaustion and acquired immune resistance, highlighting high-risk patients as potential candidates for anti-TIM-3 or anti-VISTA combination therapies. In addition, the heatmap depicting the enrichment scores of 28 immune cell types indicated elevated enrichment of certain immune cells in the high-risk group (**Figure 4C**). A total of 9 immune cell types showed differences between the two risk groups, including CD56dim natural killer cells, central memory CD8 T cells, macrophages, mast cells, monocytes, neutrophils, plasmacytoid dendritic cells, regulatory T cells, and T follicular helper cells (**Figure 4D**). Correlation analysis of differential immune cells demonstrated that macrophages were positively correlated with regulatory T cells (cor = 0.82) (**Figure 4E**). We also confirmed the correlation between different immune cells and prognostic genes (**Figures 4F–H**). PODN showed the strongest positive correlation with plasmacytoid dendritic cells (cor = 0.5), SEMA7A with T follicular helper cells (cor = 0.42), and GPR34 with T follicular helper cells (cor = 0.66). In contrast, COCH showed the strongest negative correlation with neutrophils (cor = -0.4).

### 3.5 Chromosomal localization and subcellular localization analysis

**Figure 5:**
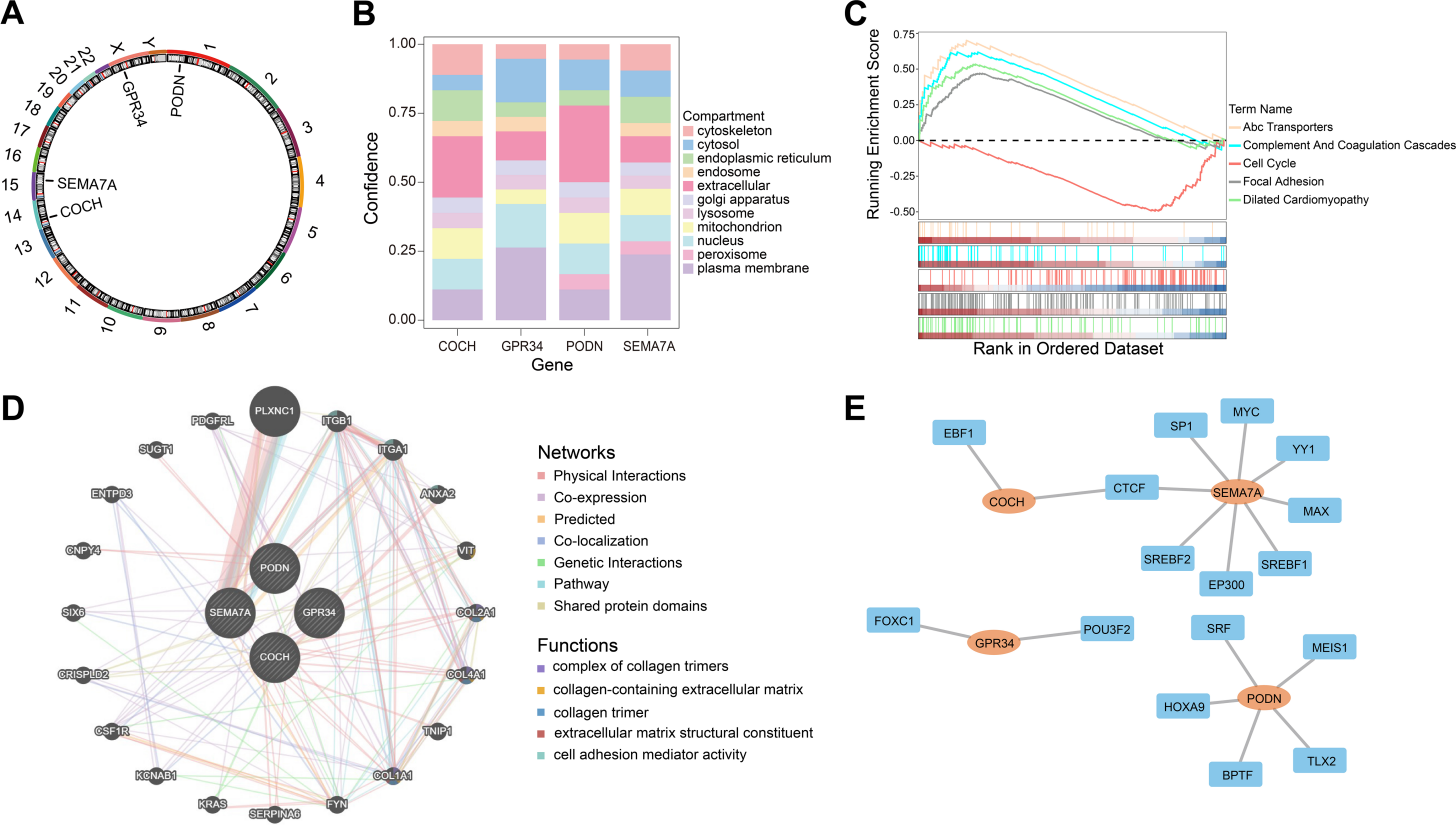
Localization analysis and functional analysis of prognostic genes. **(A)** Chromosomal localization analysis of prognostic genes. **(B)** Subcellular localization analysis of prognostic genes. **(C)** GSEA of prognostic genes. **(D)** GeneMANIA analysis of prognostic genes. **(E)** TF-mRNA network analysis of prognostic genes.

Chromosomal localization analysis indicated that PODN was located on chromosome 1, SEMA7A on chromosome 15, GPR34 on the X chromosome, and COCH on chromosome 14 (**Figure 5A**). Subcellular localization predictions suggested that the protein product of PODN was primarily localized to the extracellular region, SEMA7A and GPR34 were predominantly associated with the plasma membrane, and COCH was also mainly detected in the extracellular region (**Figure 5B**).

### 3.6 GSEA, GeneMANIA, and TF-mRNA network analysis

GSEA analysis demonstrated that the high- and low-risk group showed significant differences on 18 KEGG pathways, including ABC transporters, complement and coagulation cascades, cell cycle, focal adhesion, and dilated cardiomyopathy, among others (p < 0.05, |NES| > 1) (**Figure 5C**, **Supplementary Table 6**). The GeneMANIA analysis confirmed the top 20 interacting genes associated with the prognostic genes, including PLXNC1, ITGB1, ITGA1, ANXA2, VIT, COL2A1, COL4A1, TNIP1, COL1A1, FYN, SERPINA6, KRAS, KCNAB1, CSF1R, CRISPLD2, SIX6, CNPY4, ENTPD3, SUGT1, and PDGFRL. The GeneMANIA network indicated that the four prognostic genes are associated with multiple biological functions, such as complex of collagen trimers, collagen-containing extracellular matrix, collagen trimer, and cell adhesion mediator activity (**Figure 5D**). The TF-mRNA interaction network demonstrated that PODN was associated with five transcription factors (TLX2, SRF, MEIS1, HOXA9, and BPTF); SEMA7A was linked to eight TFs (SP1, YY1, SREBF2, SREBF1, MYC, MAX, EP300, and CTCF); GPR34 was connected to two TFs (POU3F2 and FOXC1); and COCH was associated with two TFs (EBF1 and CTCF) (**Figure 5E**).

### 3.7 Effects of SEMA7A on cell proliferation and migration of TNBC cells *in vivo* and *in vitro*

**Figure 6:**
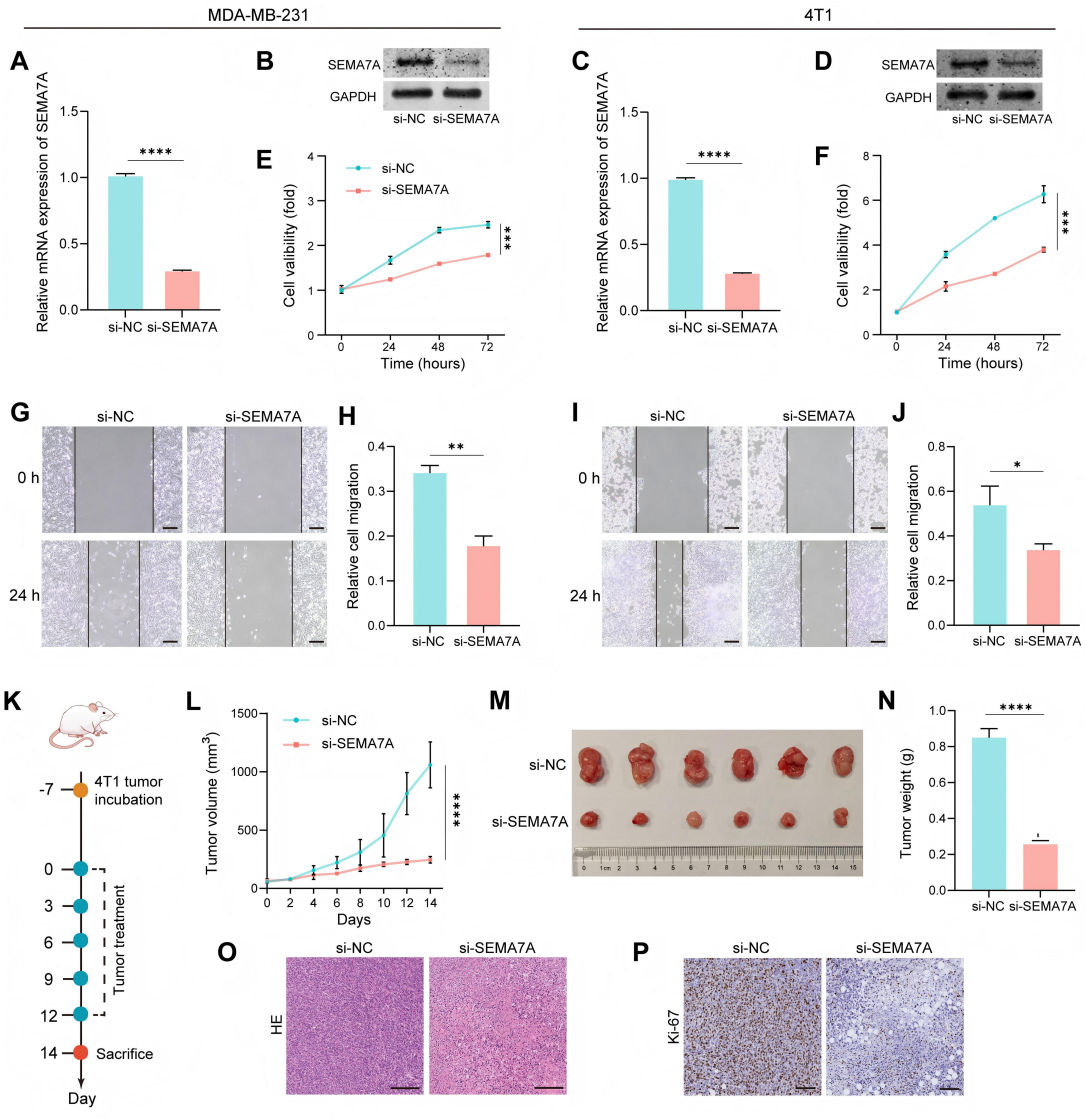
Downregulation of SEMA7A inhibits cell proliferation and migration of breast cancer cells. **(A, B)** qPCR analysis **(A)** and western blot analysis **(B)** of SEMA7A expression levels in MDA-MB-231 cells transfected with si-SEMA7A and si-NC. (n = 3). **(C, D)** RT-qPCR analysis **(C)** and western blot analysis **(D)** of SEMA7A expression levels in 4T1 cells transfected with si-SEMA7A and si-NC. (n = 3). **(E, F)** Cell proliferation analysis of MDA-MB-231 **(E)** and 4T1 **(F)** transfected with si-SEMA7A and si-NC. (n = 3). **(G, H)** Wound healing analysis **(G)** and statistical analysis **(H)** of MDA-MB-231 transfected with si-SEMA7A and si-NC. Scale bar = 100 μm. (n = 3). **(I, J)** Wound healing analysis **(I)** and statistical analysis **(J)** of 4T1 transfected with si-SEMA7A and si-NC. Scale bar = 100 μm. (n = 3). **(K)** Schematic illustration of the establishment and treatment procedure for 4T1 orthotopic breast tumor mouse model. **(L)** Tumor growth curves were plotted for the mice bearing 4T1 cells. (n = 6). **(M)** Photographs of the 4T1 orthotopic breast tumors at the endpoint of the therapeutic period. **(N)** Tumor weights of the 4T1 orthotopic breast tumors at the endpoint of the therapeutic period. (n = 6). **(O)** HE staining of 4T1 breast tumor sections. Scale bar = 200 μm. **(P)** Ki67 immunohistochemical staining of 4T1 breast tumor sections. Scale bar = 100 μm. Data are presented as the means ± SD, standard deviations. Statistical analyses were performed with Student’s t-test. *p < 0.05, **p < 0.01, ***p < 0.001, ****p < 0.0001.

Since we had previously recognized SEMA7A as the top - ranking prognostic factor, we then further examined the impact of SEMA7A in TNBC cells. *In vitro* experiments were conducted to acquire a better insight into biological function of SEMA7A. SEMA7A siRNA (si-SEMA7A) and negative control siRNA (si-NC) were transfected into both MDA-MB-231 and 4T1 cells. First, qPCR analysis and western blotting analysis consistently confirmed the SEMA7A downregulation (**Figures 6A–D**). The effects of SEMA7A on TNBC cells proliferation and migration were further validated via CCK8 and wound healing experiments. It is showed that the downregulation of SEMA7A notably suppressed both the proliferative (**Figures 6E, F**) and migratory capabilities (**Figures 6G–J**) of MDA-MB-231 and 4T1 cells. Further research was conducted on the effect of SEMA7A on tumor progression *in vivo* through an orthotopic breast tumor mice model (**Figure 6K**). It is observed that the sizes and weights of tumors in the si-SEMA7A treated group were markedly smaller than the si-NC treated group (**Figures 6L–N**). Additionally, the images of histopathological analysis of hematoxylin and eosin (HE) staining and Ki67 immunohistochemical staining of tumors in the si-SEMA7A treated group showed aberrant cellular morphology, severe necrosis and lower ki-67 level, while the si-NC treated group maintained normal cellular morphology (**Figures 6O, P**). Over all, our study confirmed that SEMA7A could promote the proliferation and migration of TNBC cells *in vitro* and promote tumor growth *in vivo*.

## 4 Discussion

TNBC is a highly aggressive subtype with bad prognosis and the conventional treatment for TNBC poses challenges due to its heterogeneity and lack of specific receptors for hormone therapy and targeted therapy. Recently, the advances in immunotherapy have shown promise in TNBC treatment and brought hopes for effective targeted therapy of TNBC (10, 31). However, a large part of TNBC patients has poor response to T cell-mediated immunotherapies. It is needed to further look into the molecular aspects of TNBC and search for new prognostic genes to anticipate the survival of TNBC patients and enhance immunotherapy efficiency. Thus, we designed a prognostic model made up of 4 TTKRGs to predict survival and immunotherapeutic responses in TNBC. Through comprehensive bioinformatic analyses, we explored the correlations with clinical and immunological characteristics, providing a novel understanding of the potential mechanisms of TNBC progression.

Four candidate genes (PODN, SEMA7A, GPR34, and COCH) were confirmed, with PODN, SEMA7A, and GPR34 identified as risk factors and COCH identified as a protective factor. Collectively, our GeneMANIA and TME analyses revealed that these genes are deeply embedded in extracellular matrix (ECM) organization and immune modulation. In the context of tumor immunology, excessive ECM deposition and stromal stiffening drive an ‘immune-excluded’ phenotype (32). Podocan (PODN), a small leucine-rich proteoglycan, and cochlin (COCH) both interact extensively with ECM components (33, 34). We postulate that PODN and COCH are critically involved in orchestrating this ‘immunosuppressive stromal activation,’ creating a dense desmoplastic barrier that physically sequesters effector T cells in the tumor stroma, thereby severely impairing T cell-mediated tumor killing (TTK). By modulating ECM stiffness, this crosstalk between tumor cells and stromal fibroblasts actively prevents CTLs from reaching the tumor parenchyma (35, 36), contributing to the poor immunotherapeutic response observed in high-risk TNBC.

Beyond the physical stromal barrier, our model highlights the formation of a robust cellular immune-tolerance network. As a member of the GPR super family, GPR34 is mainly expressed in immune cells and is involved in regulating multiple aspects of immune responses (37). Interestingly, the significant enrichment of immunosuppressive leukocyte subsets, such as M2 macrophages, regulatory T cells (Tregs), and T follicular helper (Tfh) cells in the high-risk group aligns with elevated GPR34 expression. GPR34 signaling likely facilitates the recruitment and polarization of M2 tumor-associated macrophages (TAMs) (38), which secrete immunosuppressive cytokines to suppress CD8+ T cell functionality. Furthermore, the strong positive correlation between macrophages and Tregs suggests a cooperative mechanism where TAMs and Tregs mutually sustain T cell exhaustion. The enrichment of Tfh cells further supports this immunosuppressive environment (39), indicating that the high-risk TME utilizes complex cellular crosstalk to neutralize TTK and actively reshape a microenvironment conducive to immune evasion (40–42).

Semaphorin 7a (SEMA7A) emerged as the most critical TTK-related master immune regulator in our model. SEMA7A is a neuroimmune glycoprotein and an important regulatory factor in immunopathology (43). During tumor progression, SEMA7A not only promotes invasive power but also actively regulates the TME by inducing M2 macrophage polarization and inhibiting cytotoxic T cell function (44–46). Importantly, we identified that the expression of next-generation immunosuppressive checkpoints, HAVCR2 (TIM-3) and VSIR (VISTA), was significantly upregulated in the high-risk group. Unlike the traditional PD-1/PD-L1 axis, TIM-3 and VISTA act as alternative inhibitory checkpoints that drive severe T cell exhaustion and acquired immune resistance (47). Therefore, the SEMA7A-driven TTK signature not only predicts poor prognosis but also precisely identifies a unique TNBC subpopulation that might be refractory to standard therapies but highly susceptible to emerging anti-TIM-3 or anti-VISTA combination blockades.

To validate these bioinformatics and immunological insights, we further studied the biological effects of SEMA7A in TNBC cells. CCK-8 and wound healing assays demonstrated that the downregulation of SEMA7A significantly inhibited the cell proliferation and migration capacities of MDA-MB-231 and 4T1 cells *in vitro*. Likewise, *in vivo* experiments revealed that SEMA7A downregulation strongly suppressed tumor growth, further corroborating its oncogenic nature. In summary, our studies confirmed that SEMA7A plays an essential part in TNBC progression through a dual mechanism of directly promoting the malignant phenotype of tumor cells and indirectly orchestrating the immunosuppressive microenvironment.

This study systematically explored the prognostic value and internal mechanisms of TTKRGs in TNBC. Through univariate Cox and RSF analysis, four prognostic genes (PODN, SEMA7A, GPR34, and COCH) were selected. Furthermore, a prognostic model was developed which showed good performance in the training and validation sets. Further analyses showed significant differences in clinicopathological features, drug sensitivity, tumor microenvironment, immune checkpoint expression, and immune infiltration between high and low-risk groups. GSEA suggested potential involvement in immune evasion and treatment response pathways.

Nevertheless, there may be some possible limitations in this study. Firstly, the research data are all retrospective date with limited sample sizes and inconsistent follow-up, potentially introducing selection bias. Secondly, the bioinformatic predictions lack validation through large-scale clinical cohorts, thus their clinical applicability remains to be confirmed. Besides, although our model demonstrated robust prognostic performance in both datasets, the varying optimal risk score cutoffs between the datasets reflect underlying population heterogeneity. For clinical translation, prospective validation is needed to determine whether customized or a single fixed risk score cutoffs would yield superior patient outcomes. The generalizability and stability across diverse populations and platforms require further verification. Future work should involve multi-center samples to experimentally validate the findings and elucidate the mechanisms of prediction genes, especially the function in T cell-mediated immune responses, to provide a theoretical basis for novel immunotherapy strategies.

## Data Availability

The original contributions presented in the study are included in the article/**Supplementary Material**. Further inquiries can be directed to the corresponding authors.
