# Supplementary figures and images for: Comprehensive analysis of prognostic characteristics based on T cell-mediated tumor killing related genes in triple negative breast cancer
Source: Front Immunol. 2026 Apr 10;17:1801004. doi: 10.3389/fimmu.2026.1801004 (PMC13106453; doi:10.3389/fimmu.2026.1801004)

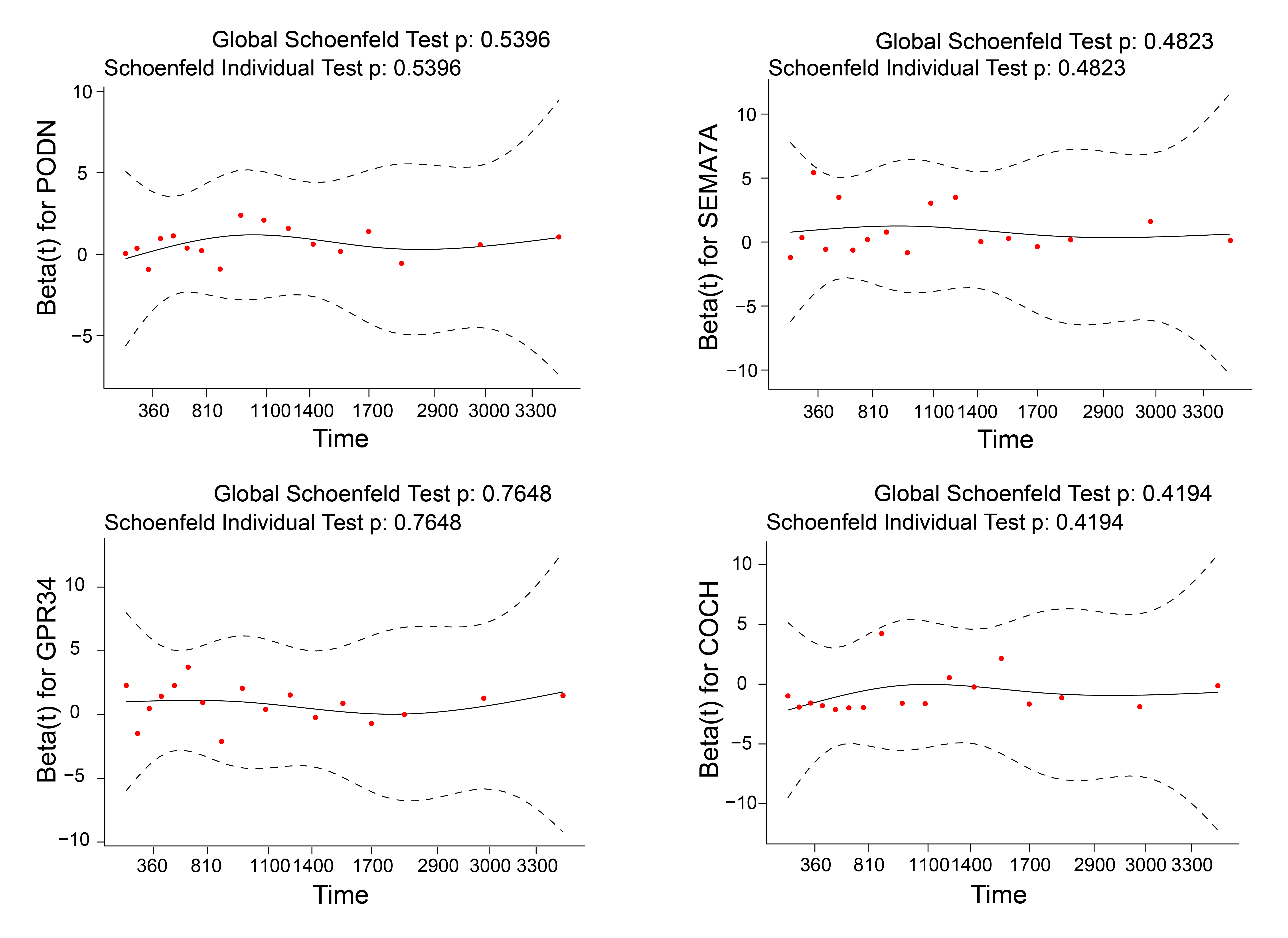

Supplement: Supplementary file 1 [file Image1.jpeg]
